# Selenoprotein W engages in overactive osteoclast differentiation in multiple myeloma

**DOI:** 10.1007/s11033-024-09517-2

**Published:** 2024-04-29

**Authors:** Hyunsoo Kim, Jiin Oh, Min Kyoung Kim, Kyung Hee Lee, Daewon Jeong

**Affiliations:** 1 Laboratory of Bone Metabolism and Control, Department of Microbiology, Yeungnam University College of Medicine, Daegu, 42415 Korea; 2 Department of Pathology and Laboratory Medicine, University of Pennsylvania School of Medicine, Philadelphia, PA19104 USA; 3 Department of Hematology-Oncology, Yeungnam University College of Medicine, Daegu, 42415 Korea; 4 Company of The Bone Science, Yeungnam University College of Medicine, Daegu, 42415 Korea

**Keywords:** Selenoprotein W, Osteoclast differentiation, Multiple myeloma, Bone disease

## Abstract

**Background:**

Patients with multiple myeloma exhibit malignant osteolytic bone disease due to excessive osteoclast formation and function. We recently identified that osteoclastogenic stimulator selenoprotein W (SELENOW) is upregulated via ERK signaling and downregulated via p38 signaling during receptor activator of nuclear factor (NF)-κΒ ligand (RANKL)-induced osteoclast differentiation. In the intrinsic physiological process, RANKL-induced downregulation of SELENOW maintains proper osteoclast differentiation; in contrast, forced overexpression of SELENOW leads to overactive osteoclast formation and function.

**Methods and results:**

We observed that SELENOW is highly expressed in multiple myeloma-derived peripheral blood mononuclear cells (PBMCs) and mature osteoclasts when compared to healthy controls. Also, the level of tumor necrosis factor alpha (TNFα), a pathological osteoclastogenic factor, is increased in the PBMCs and serum of patients with multiple myeloma. ERK activation by TNFα was more marked and sustained than that by RANKL, allowing SELENOW upregulation. Excessive expression of SELENOW in osteoclast progenitors and mature osteoclasts derived from multiple myeloma facilitated efficient nuclear translocation of osteoclastogenic transcription factors NF-κB and NFATc1, which are favorable for osteoclast formation.

**Conclusion:**

Our findings suggest a possibility that feedforward signaling of osteoclastogenic SELENOW by TNFα derived from multiple myeloma induces overactive osteoclast differentiation, leading to bone loss during multiple myeloma.

## Introduction

There is a general rule that a counterbalance between positive feedforward and negative feedback regulators determines the extent of cellular functional activity. Upregulated genes during any processing have been primarily considered as positive regulators that preferentially stimulate the processing; in contrast, downregulated genes have been thought to predominantly act as negative regulators. Dysregulation between opposite regulators induces cellular malfunction and the shift toward pathological disorders. In accordance with this concept, the relative overactivity of bone-resorbing osteoclasts compared to bone-forming osteoblasts leads to osteoporosis [1]. Mononuclear myeloid progenitors were properly differentiated into multinucleated osteoclasts by a delicate balance between receptor activator of nuclear factor (NF)-κB ligand (RANKL)-induced upregulated positive factors [for example, nuclear factor of activated T-cells, cytoplasmic (NFATc)1; osteoclast-associated, immunoglobulin-like receptor (OSCAR); dendritic cell-specific transmembrane protein (DC-STAMP); and microphthalmia-associated transcription factor (MITF)] [2–4] and downregulated negative factors [for example, inhibitor of DNA binding (Id)2, V-maf musculoaponeurotic fibrosarcoma oncogene homologue (Maf)B, interferon regulatory factor (IRF)8, and B cell lymphoma (Bcl)6] [5–8]. Interestingly, we have recently reported a novel observation that deviates from the general rule that RANKL-induced downregulated selenoprotein W (SELENOW), which contains selenocysteine encoded by a naturally occurring stop codon UGA at codon 13, stimulates osteoclast differentiation and leads to overactive osteoclast function [9]. This study showed that SELENOW-deficient and SELENOW-overexpressing mice exhibited increased and decreased bone mass due to the hypoactivity and hyperactivity of osteoclasts, respectively. Under physiological bone remodeling, SELENOW, which acts as an osteoclastogenic stimulator, is downregulated upon osteoclast differentiation and suppresses excessive osteoclast formation to maintain proper function, thereby demonstrating a negative-feedback mechanism of osteoclastogenic positive regulators.

Various destructive bone diseases such as arthritis, osteoporosis, periodontitis, and multiple myeloma are due to overactive osteoclast formation and function [10–15]. Multiple myeloma is a malignant hematological neoplasm of plasma cells, and more than 90% of patients with multiple myeloma develop destructive bone lesions, including osteopenia [16], osteolytic lesions [17], and pathologic bone fractures caused by excessive osteoclast formation and function [18]. Malignant plasma cells produce osteoclast-activating inflammatory cytokines, RANKL, macrophage inflammatory protein 1 alpha (MIP-1α), interleukin 3 (IL-3), and IL-6 [19–21]. In addition, bone marrow stromal cells surrounding multiple myeloma display noticeably increased production of osteoclastogenic cytokines, including macrophage colony-stimulating factor (M-CSF), tumor necrosis factor-α (TNF-α), IL-6, IL-11, and IL-1β [22–24]. Thus, these factors, produced by both plasma and stromal cells, stimulate osteoclast formation and function, resulting in bone destruction in the myeloma bone marrow microenvironment. In addition, comparative gene expression profiles of multiple myeloma endothelial cells revealed three upregulated genes [Bcl2/adenovirus E1B 19-kDa interacting protein 3 (*BNIP3*), immediate early response 3 (*IER3*), and *SELENOW*] [25], which potentiate over-angiogenesis involved in the consistent progression of multiple myeloma. However, there is no evidence that these genes participate in bone remodeling.

Based on the facts that ectopic expression of SELENOW induces overactive osteoclast differentiation and function, and that its expression is maintained at high levels in multiple myeloma-derived bone marrow endothelial cells, we explored whether SELENOW engages in osteoclast formation in multiple myeloma. Our findings showed that SELENOW is highly expressed in multiple myeloma patient-derived osteoclast progenitor peripheral blood mononuclear cells (PBMCs) and mature osteoclasts via feedforward signaling and may be linked to the induction of overactive osteoclast differentiation.

## Materials and methods

### Materials

TNFα (Cat# cyt-252) and IL1β (Cat# cyt-273) were purchased from ProSpec (Rehovot, Israel). PD98059 and SB203580 were from Cell Signaling Technology (Danvers, MA, USA). All other reagents were obtained from Sigma-Aldrich (St. Louis, MO, USA), unless otherwise specified.

### Peripheral blood mononuclear cell (PBMC) preparation

PBMCs were obtained from blood donors of multiple myeloma patients ranging from 59 to 84 years of age (median, 67 years) and healthy volunteers ranging from 62 to 74 years of age (median, 68.5 years) as previously described [26]. Briefly, approximately 10 mL of blood were drawn into vacutainers. Whole blood samples were subjected to density gradient centrifugation using Ficoll-Histopaque (Sigma-Aldrich) to separate PBMCs from other cells, such as erythrocytes and granulocytes, based on their differential cell densities. Collected PBMCs were washed twice with minimum essential medium-alpha (α-MEM; Hyclone, Logan, UT, USA) by centrifugation at 1,500 rpm for 5 min. To induce the monocytic macrophage-like osteoclast progenitor lineage of PBMCs, cells were cultured in α-MEM containing 10% fetal bovine serum (FBS; Hyclone) and 30 ng/mL macrophage colony stimulating factor (M-CSF) for 4 days, with fresh medium added after 2 days. This study was approved by the Institutional Review Board (IRB) (IRB no. 10-32-23) at Yeungnam University College of Medicine, and all donors provided written informed consent.

### Osteoclast differentiation

To prepare osteoclast progenitors from mice, bone marrow-derived mononuclear cells were obtained from the femur and tibia of 6-week-old C57BL/6J male mice (Central Lab Animal, Seoul, Korea) or genetically modified mice with SELENOW depletion or overexpression [9] by flushing the bone marrow cavity and centrifuging at 1,500 rpm for 5 min. The resulting cell pellet was dissolved in red blood cell lysis buffer (Sigma-Aldrich) to remove the erythrocytes. Cells were incubated with α-MEM containing 10% FBS and 5 ng/mL M-CSF overnight, and non-adherent cells were further cultured in α-MEM containing 30 ng/mL M-CSF for 2 days to generate osteoclast progenitors. Osteoclast progenitors from bone marrow cells and PBMCs were differentiated into osteoclasts in α-MEM supplemented with M-CSF (30 ng/mL) and receptor activator of nuclear factor κB ligand (RANKL; 100 ng/mL) for 4 and 6 days, respectively, with a change of fresh medium on day 2. To detect osteoclasts, cells were stained for tartrate-resistant acid phosphatase (TRAP), as described previously [27] and TRAP-positive multinucleated cells (TRAP + MNCs) with more than three nuclei were counted using a light microscope.

### Enzyme-linked immunosorbent assay (ELISA) and immunoblot analysis

For ELISA, serum was extracted from blood samples by centrifugation at 3,000 rpm and 4 ℃ for 30 min, and the levels of M-CSF (Cat# ab245714), RANKL (Cat# ab213841), IL1β ( Cat# ab214025), and TNFα (Cat# ab181421) were assessed quantitatively using ready-to-use ELISA kits (Abcam, Cambridge, MA, USA), according to the manufacturer’s protocols.

For immunoblot analysis, cells were washed twice with ice-cold phosphate-buffered saline (PBS) and lysed using radioimmunoprecipitation assay (RIPA) buffer [20 mM Tris-HCl (pH 7.5), 150 mM NaCl, 1% NP-40, 0.5% sodium deoxycholate, 1 mM EDTA, 0.1% SDS, protease inhibitor cocktail (Roche, Indianapolis, IN, USA), and phosphatase inhibitor cocktail (PhosSTOP; Roche)]. After centrifugation of the whole-cell lysates, the resulting supernatant was collected. For subcellular fractionation, cells were washed with ice-cold PBS, after which the nuclear and cytoplasmic fractions were isolated using an NE-PER™ Nuclear and Cytoplasmic Extraction Kit (Thermo Fisher Scientific, Rockford, IL, USA). To quantify the levels of specific proteins, cell lysates and nuclear and cytoplasmic fractions were separated on a 4-12% gradient SDS-PAGE (Invitrogen, Carlsbad, CA, USA), followed by immunoblot analysis. The antibodies used were as follows: NFATc1 (Santa Cruz Biotechnology, Santa Cruz, CA, USA, Cat # sc-7294), p65 (Santa Cruz Biotechnology, Cat# sc-372), SELENOW (Novus Biologicals, CO, USA, Cat# NBP1-49599), TFIIB (Abcam, Cat# ab63766), β-actin (Santa Cruz Biotechnology, Cat# 47,778), phospho-ERK (Cell Signaling Technology, Cat# 9101), ERK (Cell Signaling Technology, Cat# 9102), phospho-p38 (Cell Signaling Technology, Cat#9216), and p38 (Cell Signaling Technology, Cat#9212). Fold changes were analyzed using Image J software 1.49v (NIH, Bethesda, MD, USA).

### Reverse transcription-polymerase chain reaction

Total RNA was prepared from cells using TRIzol reagent (Invitrogen), and was used (500–2000 ng) for cDNA synthesis using SuperScript III cDNA synthesis kit (Invitrogen). The following PCR primers were used: SELENOW: (forward) 5′-CGCTTGAGGCTACAAGTCCA-3′ and (reverse) 5′-AGCAGCCACGAGAACATCAG-3′; β2M: (forward) 5′-TGAATTCACCCCCACTGAAA-3′ and (reverse) 5′-GCTCCCCACCTCTAAGTTGC-3′; TNFα: (forward) 5′-TCTCGAACCCCGAGTGACAA-3′ and (reverse) 5′-TGAAGAGGACCTGGGAGTAG-3′; IL1β: (forward) 5′-AGCCATGGCAGAAGTACCT-3′ and (reverse) 5′-CAGCTCTCTTTAGGAAGACAC-3′; NFATc1: (forward) 5′-TGTGCCGGAATCCTGAAACT-3′ and (reverse) 5′-GGCGGGAAGGTAGGTGAAAC-3′; Acp5: (forward) 5′-AACTTCCCCAGTCCCTTTCTA-3′ and (reverse) 5′-GCTGTTTCTTGAGCCAGGAC-3′; Ctsk: (forward) 5′-CCGCAGTAATGACACCCTTT-3′ and (reverse) 5′-TCAATACCCCGGTTCTTCTG-3′; and GAPDH (forward) 5′-CAAGGCTGTGGGCAAGGTCA-3′ and (reverse) 5′-AGGTGGAGGAGTGGGTGTCGC-3′.

### Luciferase assay

For assessing the promoter activity of SELENOW, the SELENOW reporter plasmid (kindly donated by Dr. Ick Young Kim, Korea University) was constructed by cloning a PCR product spanning − 973 to + 32 at the upstream position of the start codon of SELENOW into a KpnI/HindIII site of luciferase-encoding pGL3-Basic vector (Promega®, Madison, WI, USA). The reporter plasmid was transfected into RAW264.7 cells by nucleoporation according to the manufacturer’s instructions (Amaxa Biosystems, Cologne, Germany). Transfected cells were seeded in α-MEM, incubated for 12 h, and then stimulated with RANKL (100 ng/mL) or TNFα (20 ng/mL) for 24 h. To assess the promoter activity of SELENOW, cells were harvested and luciferase activity was measured using a Dual-Luciferase Reporter Assay System (Promega) and LMAX II384 luminometer (Molecular Devices, Sunnyvale, CA, USA).

### Statistical analysis

All experiments were repeated at least three times and performed in triplicates. The results are expressed as mean ± standard deviation (SD). Statistical analysis was performed using Student’s *t*-test; *P <* 0.05 was considered to be statistically significant.

## Results

### Patients with multiple myeloma show excessive osteoclast formation

**Fig. 1:**
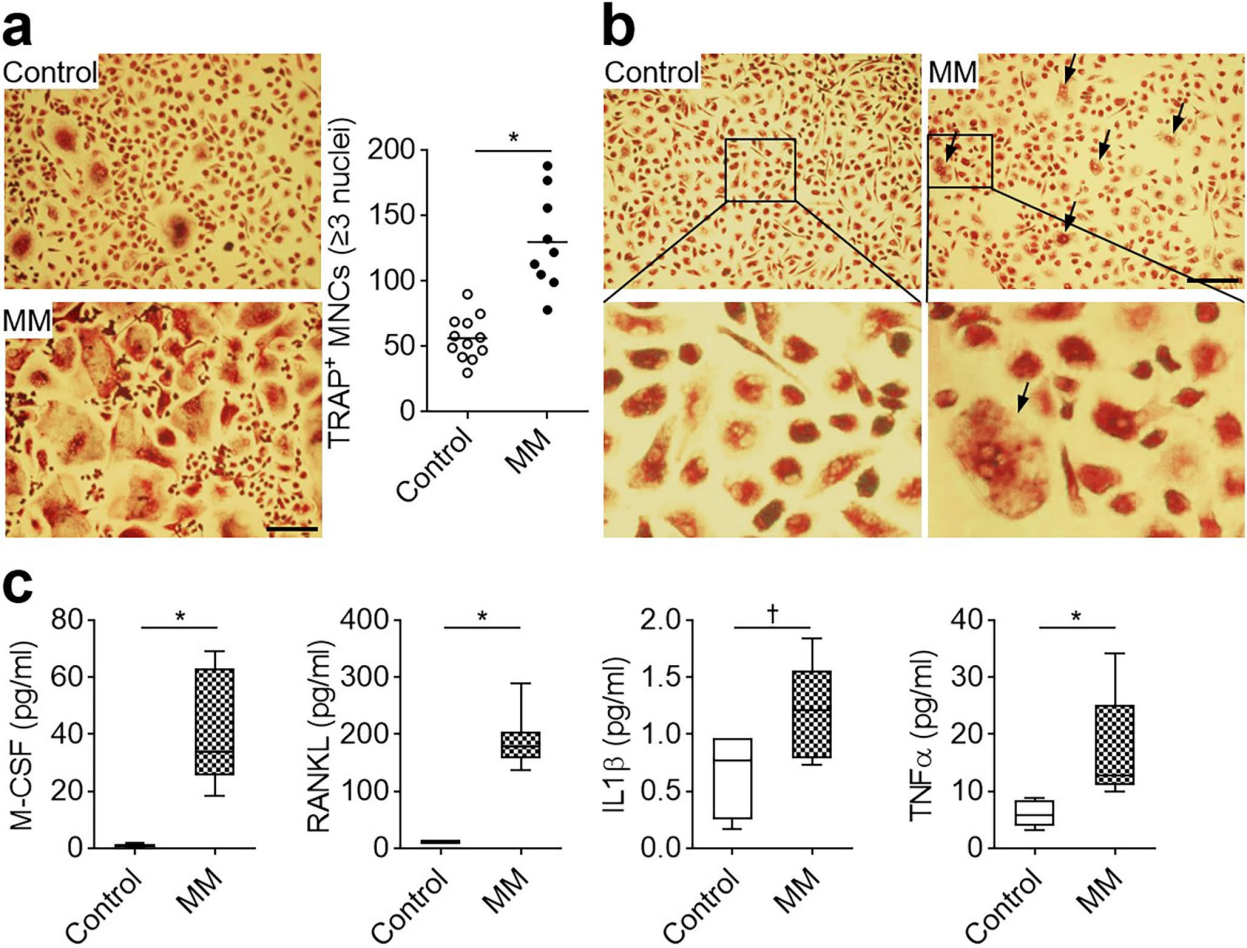
Excessive osteoclast formation in peripheral blood mononuclear cells (PBMCs) obtained from patients with multiple myeloma (MM). (**a**) Increased osteoclast differentiation in MM PBMCs. PBMCs from the healthy individuals (Control) and MM patients were differentiated into osteoclasts in the presence of M-CSF and RANKL for 6 days. Cells were stained for TRAP and TRAP^+^ MNCs (≥ 3 nuclei) were counted. Data are presented as the mean ± SD (Control, *n* = 12; MM, *n* = 9); ^*^P < 0.01. (**b**) Inherent osteoclast differentiation in MM PBMCs. To assess the extent of spontaneous osteoclast differentiation, PBMCs were cultured with M-CSF alone for 6 days and stained for TRAP. Magnified image (10×) of inset in the upper panels has been shown in the lower panels. The arrows indicate TPAP^+^ multinuclear cells. The experiment was performed in triplicate and representative images are shown. (**c**) Increased osteoclastogenic factors in the serum of MM patients. The levels of M-CSF, RANKL, IL1β, and TNFα were measured using ELISA. Data are presented as the mean ± SD (control, *n* = 5; MM, *n* = 9); ^*^P < 0.01 and ^†^*P* < 0.05. Scale bars, 200 μm

Osteoclast progenitors of monocyte/macrophage lineages mostly reside in the bone marrow and blood, and differentiate into osteoclasts. Human peripheral blood monocytes have been reported to differentiate into osteoclasts upon stimulation with M-CSF and RANKL [28]. To assess the tendency of osteoclast differentiation in multiple myeloma, we prepared PBMCs from blood obtained from healthy individuals and patients with multiple myeloma and differentiated them into osteoclasts in the presence of M-CSF and RANKL stimulation. PBMCs from multiple myeloma patients showed higher osteoclast formation than those from healthy controls (Fig. 1a). Interestingly, multiple myeloma-derived PBMCs spontaneously progressed to multinuclear osteoclast-like cells in a culture system with M-CSF alone, which could not induce osteoclast formation in PBMCs derived from healthy individuals (Fig. 1b). These results indicated that patients with multiple myeloma have a stronger inheritance of osteoclast differentiation than healthy controls. Next, we measured the levels of M-CSF and RANKL, which mediate normal osteoclast differentiation, and those of pathological osteoclastogenic inflammatory IL1β and TNFα in the serum obtained from patients with multiple myeloma using ELISA. Multiple myeloma patients were found to produce more osteoclastogenic factors (M-CSF, RANKL, IL1β, and TNFα) than healthy individuals (Fig. 1c). These results suggested that PBMCs obtained from patients with multiple myeloma possesses a higher tendency for osteoclast formation in a culture with M-CSF alone or in the presence of both M-CSF and RANKL stimulation, and a significant increase in osteoclastogenic factors was observed in patients with multiple myeloma compared to healthy individuals.

### Enhanced SELENOW expression in multiple myeloma facilitates excessive osteoclast differentiation

**Fig. 2:**
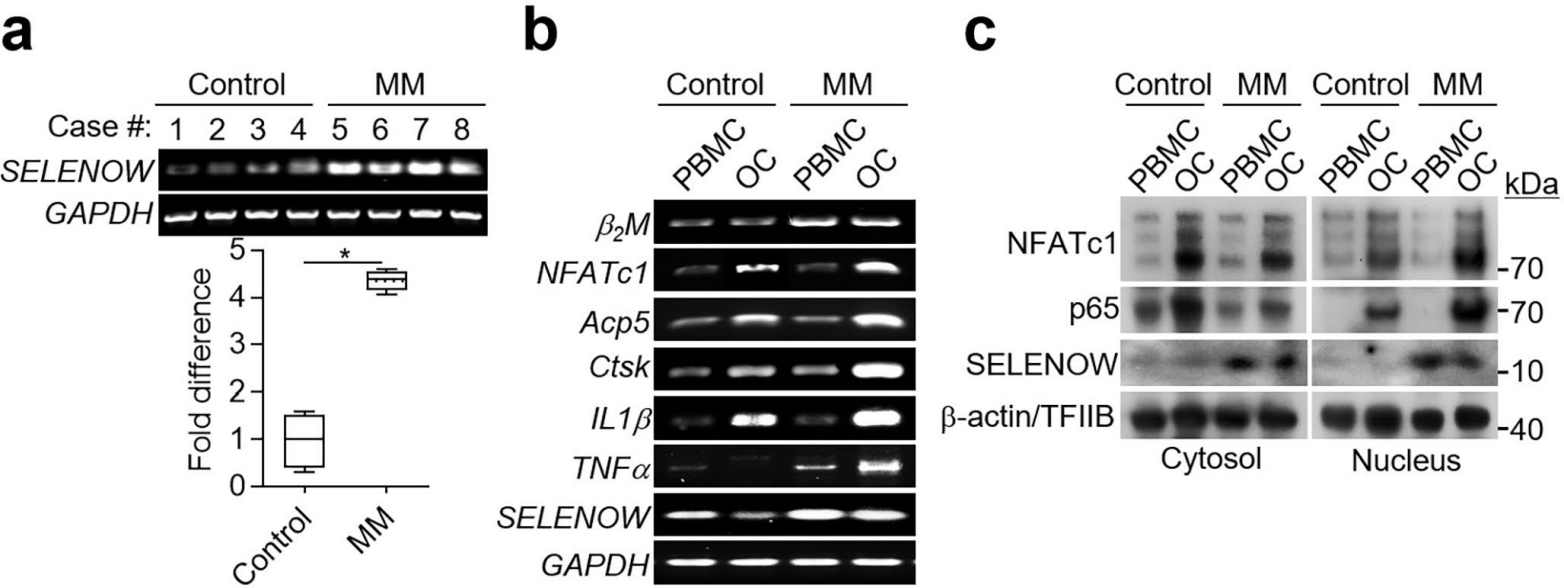
Overexpressed SELENOW in multiple myeloma (MM) activates osteoclastogenic transcription factors. (**a**) Increased mRNA level of *SELENOW* in MM-derived PBMCs. The mRNA level of *SELENOW* in PBMCs was assessed using RT-RCR and normalized to that of glyceraldehyde-3-phosphate dehydrogenase (*GAPDH*). Histogram shows the fold differences. Data are presented as the mean ± SD (*n* = 4); ^*^P < 0.01. (**b**) Increased mRNA levels of osteoclastogenic factors in MM-derived PBMCs and osteoclasts. PBMCs derived from healthy control and MM patients were differentiated in mature osteoclasts (OC) in the presence of M-CF and RANKL for 6 days. The mRNA levels of MM marker (β_2_M), inflammatory cytokines (TNFα and IL1β), osteoclastogenic factors (NFATc1, Acp5, and Ctsk), and SELENOW were measured using RT-PCR. (**c**) After osteoclast differentiation of PBMCs as in (**b**), PBMCs and osteoclasts (OC) were incubated in the absence of M-CSF and RANKL for 4 h and stimulated with 100 ng/mL RANKL for 20 min. The fractionated extracts from the nuclei and cytosol were subjected to immunoblot analysis to evaluate the extent of nuclear translocation of osteoclastogenic transcription factors (NFATc1 and p65 subunit of NF-κB) and SELENOW. The expression of β-actin (for cytosol) and TFIIB (for nucleus) was analyzed to normalize the values for individual fractions

It has been shown that multiple myeloma has osteolytic lesions caused by overactive osteoclast differentiation and activity as well as an increase in SELENOW expression in endothelial cells [25]. In addition, our previous findings suggest that SELENOW induces osteoclast differentiation by promoting the nuclear translocation of NF-κB/SELENOW or NFATc1/SELENOW cytosolic complexes [9]. In line with the previous reports, we attempted to identify a plausible connection between SELENOW expression and osteoclast differentiation in multiple myeloma. To this end, we first measured the mRNA levels of *SELENOW* in PBMCs using RT-PCR. The mRNA levels of *SELENOW* were significantly higher in multiple myeloma-derived PBMCs than in the control cells (Fig. 2a). Second, as shown in Fig. 2b, we assessed the extent of SELENOW expression during the differentiation of PBMCs into multinuclear osteoclasts with increased levels of osteoclastogenic marker genes [such as NFATc1, tartrate-resistant acid phosphatase (TRAP; Acp5), and cathepsin K (Ctsk)]. SELENOW was downregulated during osteoclast differentiation of PBMCs obtained from healthy individuals, whereas its expression was sustained during osteoclast differentiation of PBMCs obtained from patients with multiple myeloma. In addition, IL1β and TNFα, which act as pathological osteoclastogenic factors [29, 30], were highly expressed in both osteoclast progenitor PBMCs and differentiated osteoclasts from multiple myeloma patients than in those from healthy controls, which was consistent with increased levels of circulating osteoclastogenic factors in the blood obtained from patients with multiple myeloma, as shown in Fig. 1c.

**Fig. 3:**
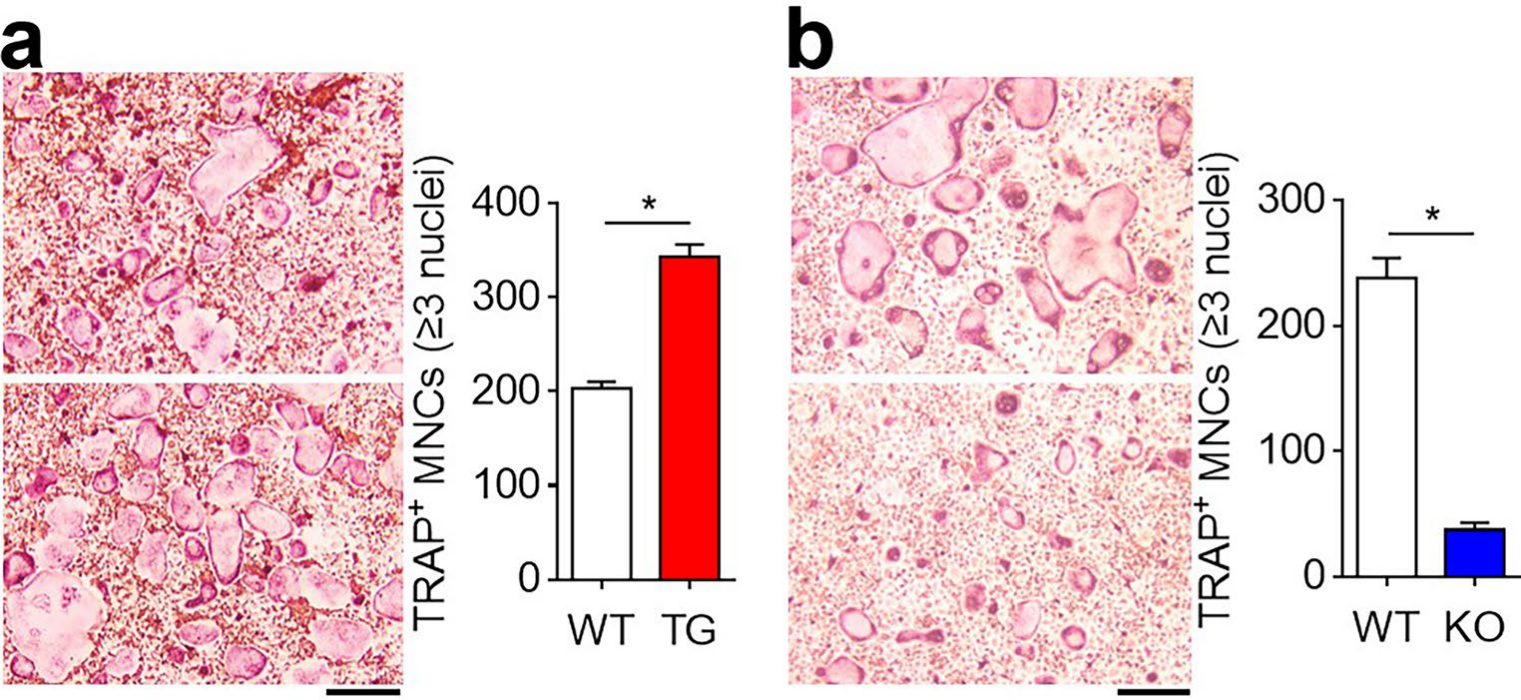
SELENOW potentiates osteoclast differentiation. (**a**) SELENOW repletion induced enhanced osteoclast differentiation. Osteoclast progenitors from wild type (WT) male littermates and age/sex-matched *SELENOW*-overexpressing transgenic (TG) mice were differentiated into osteoclasts in the presence of M-CSF and RANKL for 4 days. (**b**) SELENOW depletion induced a reduction in osteoclast differentiation. Osteoclast progenitors from wild type (WT) male littermates and age/sex-matched *SELENOW*^*−/−*^ (KO) mice were differentiated into osteoclasts as in (**a**). TRAP^+^ MNCs (≥ 3 nuclei) were counted. Data are presented as the mean ± SD obtained from triplicate experiments; ^*^P < 0.01. Scale bars, 100 μm

Osteoclastogenic SELENOW is normally repressed during osteoclast differentiation, and its repression allows physiological bone remodeling by preventing overactive osteoclast differentiation and activity [9]. Therefore, we examined whether the increased expression of SELENOW in multiple myeloma affected osteoclast differentiation. RANKL stimulation in multiple myeloma-derived osteoclasts induced elevated co-translocation of the cytosolic osteoclast-inducing essential transcription factors of NF-κB (p65 subunit) and NFATc1, and SELENOW into the nucleus as compared to that in the control cells (Fig. 2c). Based on the combined results of our previous [9] and present studies, we envisage that multiple myeloma maintains the constitutive expression of SELENOW during osteoclast differentiation and that the excess level of SELENOW facilitates nuclear translocation of NF-κB and NFATc1, resulting in excessive osteoclast formation. We observed that SELENOW-overexpressing and SELENOW-deficient osteoclast progenitors showed an increase and a decrease in osteoclast formation, respectively, compared to control cells (Fig. 3). Consequently, these results indicated that overexpression of SELENOW in multiple myeloma may be associated with excessive osteoclast differentiation via efficient nuclear translocation of the osteoclastogenic transcription factors NF-κB and NFATc1.

### TNFα induces excessive osteoclast differentiation by sustaining SELENOW expression

**Fig. 4:**
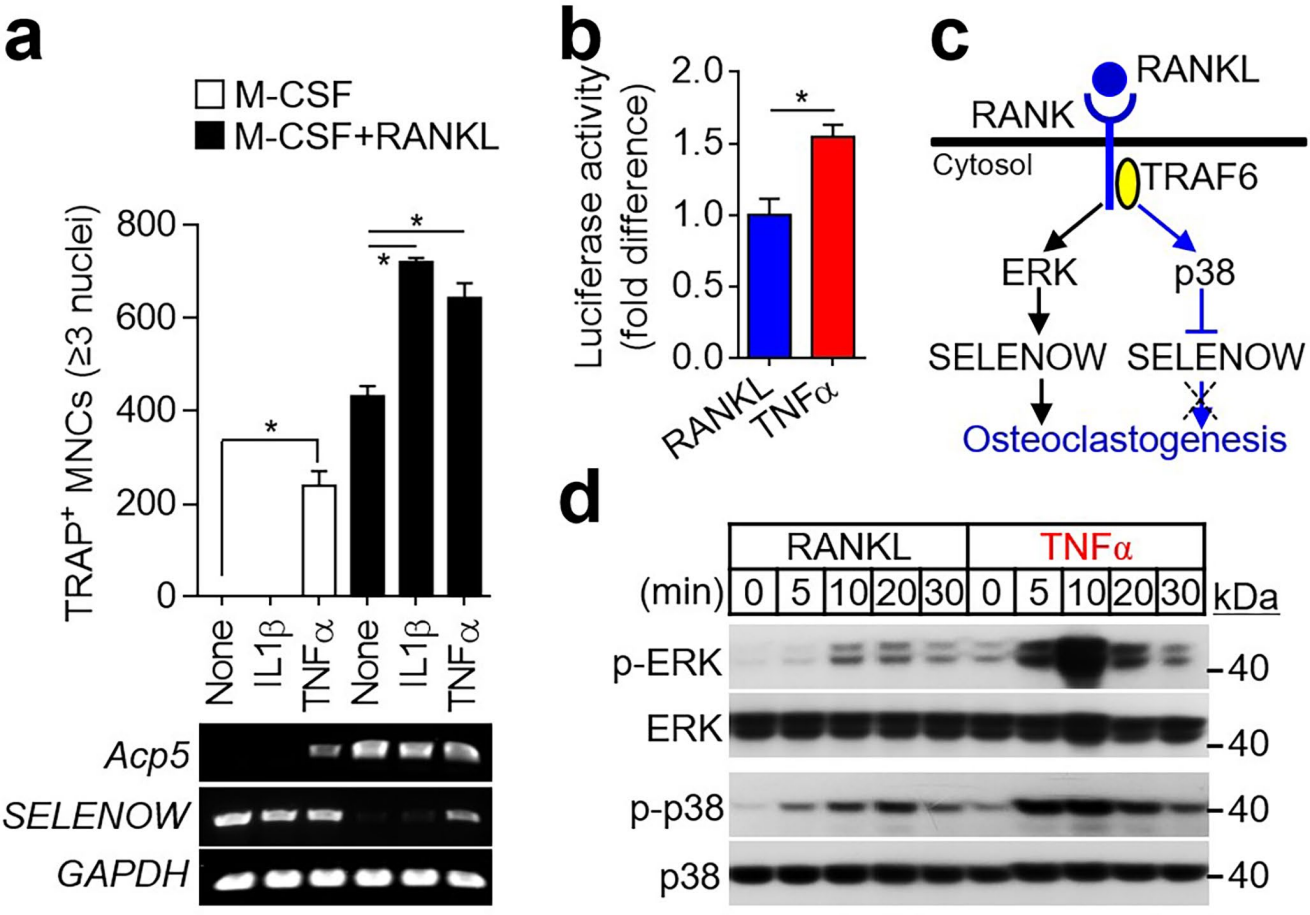
TNFα, a pathological osteoclastogenic factor in multiple myeloma, stimulates SELENOW expression by activating ERK signal. (**a**) Increased SELENOW expression by TNFα. Osteoclast progenitors were cultured in a medium containing M-CSF alone or M-CSF and RANKL in the absence or presence of IL1β (10 ng/mL) or TNFα (20 ng/mL) for 4 days. TRAP^+^ MNCs (≥ 3 nuclei) were counted. In addition, the mRNA level of *SELENOW* was analyzed using RT-PCR. *Acp5* and *GAPDH* were used as an osteoclast-specific gene and housekeeping gene, respectively. Data are presented as the mean ± SD obtained from triplicate experiments; ^*^P < 0.01. (**b**) Reporter plasmid encoding luciferase under SELENOW promoter was transfected into RAW264.7 cells and then treated with RANKL (100 ng/mL) and TNFα (20 ng/mL) for 24 h. The luciferase activity was measured using a luminometer. Data are presented as the mean ± SD obtained from triplicate experiments; ^*^P < 0.01. (**c**) Schematic diagram for signaling circuit that regulates SELENOW expression during osteoclast differentiation. (**d**) TNFα induces ERK activity to a higher extent than that induced by RANKL. Osteoclast progenitors were incubated without M-CSF for 4 h and stimulated with RANKL (100 ng/mL) or TNFα (20 ng/mL) for the indicated times. The extent of ERK and p38 activation was visualized using immunoblots with specific antibodies

Considering that SELENOW is highly expressed in multiple myeloma and that it promotes osteoclast differentiation, we analyzed the molecular mechanisms underlying SELENOW upregulation in multiple myeloma with an increase in circulating osteoclastogenic factors, including M-CSF, RANKL, IL1β, and TNFα. When osteoclast differentiation was induced by M-CSF and RANKL in combination with IL1β or TNFα, TNFα promoted RANKL-induced osteoclast differentiation (Fig. 4a, column 6). Moreover, TNFα induced osteoclast formation during RANKL-independent osteoclast differentiation (Fig. 4a, column 3). Notably, whereas SELENOW was downregulated during physiological osteoclast differentiation without TNFα (Fig. 4a, column 4), a certain level of SELENOW was maintained during the excessive osteoclast formation in the presence of TNFα (Fig. 4a, columns 3 and 6). Because this phenomenon did not respond to IL1β, we did not further study it in the context of IL1β-mediated SELENOW regulation. A luciferase reporter assay showed that SELENOW promoter activity was significantly increased following TNFα stimulation compared to RANKL stimulation (Fig. 4b). These results indicated that TNFα induces a stronger expression of SELENOW than RANKL.

When considering osteoclast metabolism with normal physiological bone remodeling, SELENOW is known to be upregulated via RANKL-induced ERK activation and downregulated via RANKL-induced TRAF6-dependent p38 activation (Fig. 4c) [9]. To analyze the role of ERK and p38 signaling in TNFα-induced SELENOW upregulation, we compared the extent of ERK and p38 activation induced by RANKL and TNFα. As shown in Fig. 4d, TNFα stimulation induced stronger and longer-lasting activation of ERK and p38 than RANKL stimulation. When comparing ERK and p38 activities, TNFα induced greater activation of ERK than that of p38. Thus, this showed that greater TNFα-induced activation of ERK than p38 renders constitutive expression of SELENOW.

### The seesaw balance between ERK and p38 signaling modulates SELENOW expression

**Fig. 5:**
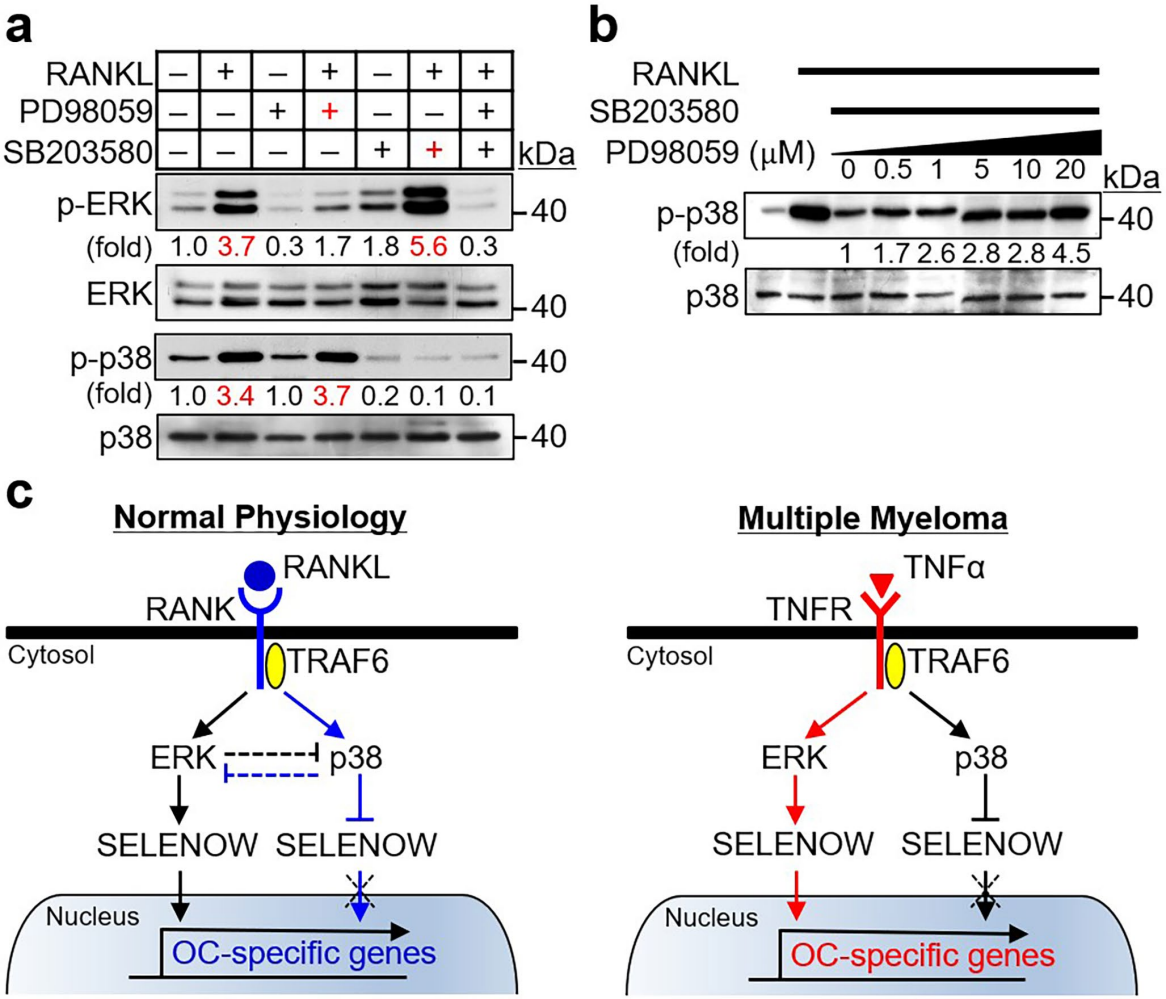
Seesaw balance between ERK and p38 signaling modulates SELENOW expression. (**a**) The inactivation of p38 induced increased ERK activation. Osteoclast progenitors were incubated without M-CSF for 4 h, pretreated with a selective inhibitor of ERK (PD98059; 20 µM) and/or p38 (SB203580; 20 µM) for 30 min, and then stimulated with RANKL (100 ng/mL) for 10 min followed by immunoblotting with specific antibodies. (**b**) The inactivation of ERK induced increased p38 activation. Osteoclast progenitors were incubated without M-CSF for 4 h and pretreated with p38 inhibitor (SB203580; 5 µM) and ERK inhibitor (PD98059; ranging from 0 to 20 µM) for 30 min. Then the cells were stimulated with RANKL (100 ng/mL) for 10 min followed by immunoblotting with specific antibodies. The fold change for specific bands was analyzed using an image analyzing software. (**c**) The proposed signaling route for differential expression control of SELENOW under physiological and multiple myeloma conditions

As SELENOW expression is regulated based on the strength of ERK and p38 activity, we investigated whether a crosstalk exists between ERK and p38 signaling. To address this, we examined whether p38 inactivation affected the strength of ERK activity and vice versa. When comparing ERK and p38 activation in response to RANKL, treatment with a specific inhibitor of p38 (SB203580; 20 µM) led to enhanced RANKL-induced activation of ERK compared to RANKL alone (Fig. 5a, line 2 versus line 6), indicating that p38 repression accelerates ERK activation. Conversely, the inhibitor of ERK (PD98059; 20 µM) promoted RANKL-induced activation of p38; however, this effect was ambiguous (Fig. 5a, line 2 versus line 4). To clarify p38 activation upon ERK inhibition, we attempted to reduce the basal activity of p38 by treating osteoclast progenitors with a low concentration (5 µM) of p38 inhibitor. As shown in Fig. 5b, RANKL-induced p38 activation was gradually increased in a concentration-dependent manner by the ERK inhibitor, indicating that ERK suppression promotes p38 activation. These data showed that RANKL-induced ERK and p38 activation in osteoclast progenitors is controlled by a counterbalance between the two signals.

## Discussion

The recent therapeutic drugs for multiple myeloma are proteasome inhibitors (e.g., bortezomib, carfilzomib, and ixazomib), immunomodulatory agents (e.g., thalidomide, lenalidomide, and pomalidomide), monoclonal antibodies (e.g., daratumumab, isatuximab, and elotuzumab), and B-cell maturation antigen-targeting immunotherapies such as bispecific antibodies, antibody-drug conjugates, and chimeric antigen receptor T-cell therapies [31, 32]. Since the potent and specific inhibitors of ERK signaling cascade have been shown to be effective for the treatment of various cancers including ovarian, breast, and pancreatic cancer [33], SELENOW, which is constitutively expressed via ERK-mediated feed-forward signaling, may be served as a possible therapeutic target for the drug development of multiple myeloma with osteolytic lesions. Hence, whether suppressing the constitutive expression of SELENOW, an osteoclastogenic stimulator, is effective for multiple myeloma treatments will need further pre-clinical and clinical evaluation of specific ERK mitogen-activated protein kinase inhibitors.

We propose the role of SELENOW in osteoclast differentiation. SELENOW, an osteoclastogenic stimulator, is upregulated and downregulated via TRAF6-independent ERK and TRAF6-dependent p38 activation during RANKL-induced osteoclast differentiation, respectively, and its expression is regulated by a seesaw-like counterbalance between the two signaling pathways (Fig. 5c, left panel). In normal physiological bone remodeling, RANKL-induced repression of osteoclastogenic SELENOW induces proper osteoclast formation and blocks osteoporosis caused by overactive osteoclasts [9]. In contrast, multiple myeloma that is associated with osteolytic bone disease due to excessive osteoclast formation and activity has high levels of circulating pathological inflammatory cytokine TNFα in the patient blood as well as SELENOW in osteoclast progenitors and mature osteoclasts. The large amount of osteoclastogenic TNFα in multiple myeloma may be conferred a constant expression of SELENOW during RANKL-induced osteoclast differentiation. In conclusion, TNFα-induced constitutive expression of SELENOW may be due to stronger positive-feedforward signaling of ERK than negative feedback signaling of p38. As a result, upregulation of the osteoclastogenic stimulator SELENOW may be involved in osteoporotic disease due to overactive osteoclast differentiation and function (Fig. 5c, right panel). As SELENOW is differentially regulated under physiological and pathological conditions, further studies are needed to identify the role of SELENOW in various bone defects, including postmenopausal osteoporosis [34], arthritis-causing bone deformity [35], and bone-metastatic cancer-mediated osteoporosis [36]. Our findings suggest a novel signaling circuit underlying the stimulatory action of SELENOW in osteoclast differentiation in multiple myeloma with osteolytic lesions.

## Data Availability

No datasets were generated or analysed during the current study.
